# Depression Screening and Treatment Among Nonpregnant Women of Reproductive Age in the United States, 1990-2010

**Published:** 2011-10-15

**Authors:** Sherry L Farr, Patricia M. Dietz, Falicia A. Gibbs, Jessica R. Williams, Stephen Tregear

**Affiliations:** Division of Reproductive Health, National Center for Chronic Disease Prevention and Health Promotion, Centers for Disease Control and Prevention; National Center for Chronic Disease Prevention and Health Promotion, Centers for Disease Control and Prevention, Atlanta, Georgia; National Center for Chronic Disease Prevention and Health Promotion, Centers for Disease Control and Prevention, Atlanta, Georgia; Manila Consulting Group, McLean, Virginia; Manila Consulting Group, McLean, Virginia

## Abstract

**Introduction:**

Whether routine screening for depression among nonpregnant women of reproductive age improves identification and treatment of the disorder remains unclear. We conducted a systematic review of the literature to address 5 key questions specific to this population: 1) What are the current national clinical practice recommendations and guidelines for depression screening; 2) What are the prevalence and predictors of screening; 3) How well do screening tools detect depression; 4) Does screening lead to diagnosis, treatment, and improved outcomes; and 5) What are the most effective treatment methods?

**Methods:**

We searched bibliographic databases for full-length articles published in English between 1990 and 2010 that addressed at least 1 of our key questions.

**Results:**

We identified 5 clinical practice guidelines pertinent to question 1, and 12 systematic reviews or post-hoc analyses of pooled data that addressed questions 3 through 5. No systematic reviews addressed question 2; however, we identified 4 individual studies addressing this question. Current guidelines do not recommend universal screening for depression in adults, unless staff supports are in place to diagnose, treat, and follow up patients. Reported screening rates ranged from 33% to 84% among women. Several validated screening tools for depression exist; however, their performance among this population is unknown. Screening in high-risk populations may improve the patient's receipt of diagnosis and treatment. Effective treatments include exercise, psychotherapy, and pharmacotherapy.

**Conclusion:**

More research is needed on whether routine screening for depression among women of reproductive age increases diagnosis and treatment of depression, improves preconception health, and reduces adverse outcomes.

## Introduction

Approximately 14.8 million US adults (6.7%) experience major depression in a given year (1), and women are 1.7 times as likely to experience depression as men (2). The 12-month prevalence of major depressive disorder, 1 form of clinical depression, for nonpregnant women aged 18 to 50 ranges from 8% to 16% (2,3) and may be highest among low-income women, for whom prevalence of depression (defined as minor depression/dysthymia or major depression) is estimated at 29% (4). Yet, only half of women with depression have ever received a clinical diagnosis of the disorder (4), which is the first step toward treatment and recovery. Major depression is the leading cause of disability in the United States for adults aged 15 to 44 (5) and is associated with diabetes (6), stroke, and coronary heart disease (7). Depression affects a woman's preconception health (8); depression during pregnancy may affect pregnancy outcomes, such as preterm delivery and low birth weight (9-11) and may adversely affect the child's intellectual development, behavior, and mental health (12). In 1 study among women enrolled in Kaiser Permanente Northwest, approximately half of women with clinically diagnosed perinatal depression experienced depression in the 39 weeks before pregnancy (13), highlighting the need for identification and treatment of depression among nonpregnant women of reproductive age.

Nonpsychiatric clinicians have difficulty recognizing depression in their patients (14,15). One meta-analysis found that nonpsychiatric clinicians accurately diagnosed depression in only 36% of depressed patients; another meta-analysis found a rate of 47% (14,15). However, it remains unclear whether routine screening for depression among nonpregnant women of reproductive age, defined as aged 15 to 44 years, improves identification, increases treatment rates, and leads to better overall and preconception health. To consolidate current knowledge in this area, we conducted a systematic review of the literature to address 5 key questions specific to this group of women: 1) What are the current national clinical practice recommendations and guidelines for depression screening; 2) What are the prevalence and predictors of screening; 3) How well do screening tools detect depression; 4) Does screening lead to diagnosis, treatment, and improved outcomes; and 5) What are the most effective treatment methods?

## Methods

### Data sources

We searched the following electronic bibliographic databases: PubMed/MEDLINE (www.ncbi.nlm.nih.gov/pubmed/), PsycINFO (www.apa.org/pubs/databases/psycinfo/index.aspx), the Cochrane Database of Systematic Reviews (Cochrane Reviews)(www2.cochrane.org/reviews/); and the National Guideline Clearinghouse (http://www.guideline.gov/). We used a combination of free-text terms and terms from the National Library of Medicine's Medical Subject Headings (MeSH) (www.nlm.nih.gov/mesh/). We conducted 3 separate searches focused on the following: 1) screening guidelines, 2) screening for depression in nonpregnant women of reproductive age, and 3) treatment for depression. We limited searches to English-language articles published between January 1, 1990, and December 1, 2010, that described studies enrolling human participants. Free-text terms included those related to screening and treatment of depression, population-based surveys, and study types (Appendix). We supplemented searches of bibliographic databases by reviewing reference lists of retrieved articles and by searching reports, studies, articles, and monographs produced by federal and local government agencies, private organizations, and educational organizations. For searches of articles, we used a 2-stage approach. We first searched for systematic reviews, defined as articles that synthesized relevant data from a number of independent studies and included well-defined, comprehensive search strategies, and then for individual studies, defined as research studies involving human subjects that were not meta-analyses or systematic reviews.

### Study selection

We developed inclusion criteria for each question a priori (Table 1). We conducted searches between March and December 2010.

For question 1, we initially identified depression screening guidelines from 190 national organizations. After a review of summaries of the guidelines, we deemed 21 relevant and reviewed them fully. Of these, we excluded 16 because they were not from US-based organizations or because the guidelines did not focus primarily on depression.

For study questions 2 through 5, an initial search of systematic reviews, meta-analyses, and post hoc analyses of pooled data found 5,200 mentions of depression. From this initial search, we categorized these mentions by each study question. We reviewed some studies for more than 1 study question. The reasons for exclusion were that a separate synthesis of women was not reported (n = 186); the study was not a systematic review, meta-analysis, or post-hoc analysis of pooled data (n = 43); or the study did not address our study question (n = 105). For questions 2 through 5, the initial searches found 2,337 potential individual studies. We reviewed 334 individual studies and excluded 330 because they did not address our study questions. Therefore, we include a total of 12 systematic reviews, meta-analyses, or post-hoc analyses of pooled data and 4 individual studies that met our inclusion criteria for questions 2 through 5. Below, we summarize specific studies included in each key question.

For question 5, we limited nonpharmacotherapy treatments to treatments described by the greatest number of systematic reviews identified: exercise and psychotherapy. We also limited pharmacotherapy to the 2 most common types, selective serotonin reuptake inhibitors (SSRIs) and selective norepinephrine reuptake inhibitors (SNRIs). For pharmacotherapy, we included only studies published from January 1, 2000, to December 1, 2010, because medications change over time, recent studies are most relevant, and systematic reviews offer a succinct summary measure of effectiveness.

### Data extraction

Data extracted from the selected studies included study populations, outcomes and covariates, and results. For all study questions, at least 2 authors reviewed and discussed each article until both authors concurred on its inclusion.

## Results

We identified recommendations and guidelines from 5 national groups to answer question 1, and we identified 16 studies that met our final review criteria for questions 2 through 5. For question 2, we found 4 individual studies; for question 3, 1 meta-analysis; for question 4, 2 meta-analyses and 1 systematic review; and for question 5, 5 meta-analyses and 3 post-hoc analyses of pooled data.

**Question 1. What are the current national clinical practice recommendations and guidelines for depression screening?**

We identified recommendations and guidelines from 5 national groups for screening adults for depression (16-20); however, none provided specific guidance for nonpregnant women of reproductive age (Table 2). The US Preventive Services Task Force (USPSTF) recommends that providers screen adults for depression only when staff-assisted depression care supports are in place to ensure accurate diagnosis, effective treatment, and follow-up (17). The USPSTF defines "staff-assisted care supports" as clinical staff who assist the primary care clinician by providing some direct depression care or coordination, case management, or mental health treatment. The American Academy of Family Physicians follows USPSTF guidelines for screening all adult patients for depression, using the clinician's choice of screening method (19). The US Department of Veterans Affairs (VA) recommends annual depression screening for adult patients in primary care but notes the USPSTF recommendation of screening only when staff-assisted supports are in place (18). The American College of Obstetricians and Gynecologists (ACOG) does not advocate for or against screening for depression during well-woman care, but states that when clinicians identify a woman with depression, they must provide follow-up care if they do not refer her for care elsewhere (16). The American Academy of Pediatrics (AAP) recommends screening mothers for depression at the 1-, 2-, 4- and 6-month well-child visits and beyond the postpartum period (20). The American Psychiatric Association has no published guidelines specific to depression screening, but in its guidelines on treatment for major depressive disorder, it acknowledges that primary care physicians, obstetricians, and physicians of other disciplines may screen for depression and initiate treatment for patients (21). The USPSTF, VA, ACOG, and AAP mention the 2-item Patient Health Questionnaire (PHQ-2), as an example of a short, standardized screener for depression.

**Question 2. What are the prevalence and predictors of screening?**

We did not find any studies that assessed prevalence and predictors among nonpregnant women of reproductive age only. Four individual studies reported on prevalence rates of screening for depression among adult women of any age in primary care settings (22,23) or by providers serving women of reproductive age in obstetric practices (24,25). Desai et al evaluated the VA experience in implementing universal screening, using a validated instrument, for depression (22). This study included 21,000 people receiving care at VA facilities across the United States in 2002. More than 84% of women of any age and almost 80% of women and men aged 45 or younger were screened for depression. Among patients of all ages, no difference was found in screening rates between women and men, but patients were less likely to be screened if they were younger, unmarried, had greater service disability, or had medical comorbidity. 

Tudiver et al examined screening rates for 615 adult women aged 21 to 89 years accessing primary care in 2003 at 19 rural health clinics in the United States (23). Rates of screening were low; 2.4% of visits documented formal screening (ie, through use of a validated instrument), and 33.2% of visits documented informal screening (ie, depression questions noted without mention of a screening tool).

Two studies examined the prevalence of screening for depression among obstetrician-gynecologists (OB/GYNs) (24,25). LaRocco-Cockburn et al surveyed practicing OB/GYNs in Washington State in 2001 (25). Of the 282 (56%) who returned the survey, 44% reported often or always screening for depression in patients, 41% reported screening sometimes, and 15% reported never screening for depression regardless of signs or symptoms. In this study, 81% of OB/GYNs used their own questions about mood or mental health, 32% used a validated screening tool (specific tool not reported), 16% used a validated patient self-report paper-and-pencil test, and 7% used a structured clinical interview.

Dietrich et al conducted a cross-sectional survey among 437 randomly selected US OB/GYNs (response rate, 58.3%) who had completed residency training in the previous 5 years and currently provided care (24). Approximately 40% of recent graduates and 50% of residents reported that depression was included on their practice encounter form. Only 9% to 12% reported routinely asking about depression or using a screening questionnaire to identify major or minor depression. The most common reasons for recognition of depression by OB/GYNs were that the patient appeared distressed (38%), presented with a symptom (34%), or introduced the topic directly (26%).

**Question 3. How well do screening tools detect depression?**

We did not find any studies that assessed performance of depression screening tools among nonpregnant women of reproductive age only. Several short (1-2 questions) and longer (up to 30 questions) validated tools are available to screen for depression in primary care populations (26,27). The PHQ-2, which asks, “Over the past 2 weeks, how often have you been bothered by a) little interest or pleasure in doing things and b) feeling down, depressed, or hopeless,” may perform as well as longer tools (28) or could be used as an initial screen, with a longer screen administered to patients with affirmative answers to both questions (18). Depression-specific instruments may help clinicians recognize depression more easily than instruments that measure multiple mental health conditions (29). With any screening tool, a diagnostic interview is needed to confirm the presence of depression.

We found 1 meta-analysis of the performance of instruments among adult men and women in primary care settings (30). It examined performance of case-finding instruments used for routine screening in primary care populations of men and women of all ages (30). This article evaluated only 21 of the 38 studies identified (because of study limitations); the 21 studies examined 16 validated case-finding instruments. For detecting major depression in a primary care population using routine screening with a validated case-finding tool, the median sensitivity was 85% (range, 50%-97%), the median specificity was 74% (range, 51%-98%), and no statistically significant differences between instruments were found. The study concluded that several case-finding instruments are feasible to use in primary care settings and that the instruments perform sufficiently to facilitate identification of depression.

**Question 4. Does screening lead to diagnosis, treatment, and improved outcomes?**

We did not find any systematic reviews or individual studies that addressed this question among nonpregnant women of reproductive age only. We found 2 meta-analyses (29,31) and 1 systematic review (32) that examined this question among the general population in primary care and hospital settings. Two studies (31,32) were conducted to inform and update USPSTF guidelines on screening for depression in adults. Pignone et al reviewed randomized trials published between January 1994 and August 2001 and included 14 studies in primary care settings that examined the effect of screening patients for depression on identification, treatment, and health outcomes (31). The included trials examined a range of screening intervention strategies, including feedback of screening scores, feedback and general education of providers, feedback and treatment advice, and integrated recognition and management approaches with coordinated follow-up of diagnosis and treatment. Screening resulted in a 2- to 3-fold increase in clinicians' recognition of depression. However, in a comparison of absolute differences in proportions treated, the effect of screening on rates of treatment was mixed; 4 studies found positive effects and 5 studies found no effect. Increases in rates of treatment generally resulted in increases in prescriptions for antidepressants rather than referrals to mental health professionals. Three out of 7 studies included in the meta-analysis found significant improvement in depression between groups screened for depression and groups not screened. The meta-analysis found that patient and provider characteristics, use of particular outcome measures, follow-up time, or trial quality did not explain the mixed findings; however, insufficient power may explain the results of some negative trials. A meta-analysis of the 7 studies showed that screening with or without further intervention was associated with a 13% (95% confidence interval [CI], 5%-21%) reduction in risk of remaining depressed. Additionally, variations in interventions limited their interpretation of findings.

O'Connor et al (32) published an update to the study by Pignone et al. The update included studies published from January 1998 to December 2007 on randomized controlled trials (RCTs) conducted in primary care settings among the general adult population. The authors found 2 good-quality and 2 fair-quality RCTs not included in the study by Pignone et al. The updated review supported the original findings, that primary care depression screening may be effective when the treating physician works with other staff who provide part of the depression care, such as assessment and monitoring, or when extra efforts are made to enroll patients in mental health specialty care.

The study by Gilbody et al (29) examined the effect of screening using a standardized depression screening or outcome assessment instrument alone, without substantial organizational enhancements (such as clinician education, nurse case management, and integration between primary and secondary care), on recognition of depression and improvement in outcomes among nonpsychiatric patients in primary care and hospital settings. The study identified 16 RCTs comparing usual care with routine screening administered by research staff and feedback of results to clinicians. The RCTs did not report effects separately among women. Eleven of the 16 examined the effect of screening on the clinician's recognition of depression. Seven of the 16 were conducted among the general population and found screening was not associated with increased likelihood of recognition of depression (relative risk [RR], 1.03, 95% CI, 0.85-1.24). In 4 trials conducted among high-risk populations, screening increased the likelihood of recognition of depression by 67%. Ten of the 16 studies examined the effect of depression screening on management of depression. Screening marginally increased the likelihood of the patient receiving any intervention for depression (RR, 1.30, 95% CI, 0.97-1.76) with no difference between high-risk and general primary care populations.

In pooled data from 5 studies that examined the effect of screening on depression outcomes, the meta-analysis found no effect (standardized mean difference, −0.02; 95% CI, −0.25 to 0.20) (29). The authors conclude that use of screening instruments alone in unselected populations within primary care and hospital settings, without organizational enhancements, does not improve rates of depression treatment or outcomes. They state that routine screening among high-risk populations may be more effective.

**Question 5. What are the most effective treatment methods**

We found 5 meta-analyses and no individual studies that met the inclusion criteria for nonpharmacologic treatments and 3 post-hoc analyses of pooled data that examined the effectiveness and safety of SSRIs and SNRIs in women.

Three meta-analyses explored the association between exercise and depression and evaluated differences by sex (33-35) (Table 3). All found that exercise reduced mean depression scores (effect size [ES], 0.53-0.80) with no difference by sex. North et al (35) reviewed 80 studies of any design, 16 of which included women only. Exercise was defined as aerobic exercise and muscular strength-building. The overall ES was −0.53. Craft et al (33) included 30 studies of any design, 4 that included women only and included aerobic and resistance exercise. They found a greater reduction in depression symptoms among subjects with more severe depression and those undergoing longer interventions.

In the third meta-analysis, Rethorst et al (34) reviewed 58 published RCTs, 7 of which included women only, and evaluated moderate to vigorous exercise as a treatment for depression. Controls either received no treatment or were on a wait list for treatment. Exercise was found to be beneficial in studies of participants clinically diagnosed with depression (17 studies) and among participants not clinically diagnosed with depression (40 studies), although the ES was greater among clinically diagnosed samples (ES, −1.03 for clinically diagnosed and ES, −0.59 for not clinically diagnosed).

We found 2 meta-analyses (36,37) that evaluated different types of psychotherapy (cognitive therapy [CT], individual versus group psychotherapy, and short-term psychodynamic psychotherapy) by sex (Table 3). Overall, the meta-analyses consistently found psychotherapy more effective than no treatment. Robinson et al (37) analyzed 58 studies published during 1976 through 1986, 29 of which focused on depression outcome measures. The combined sample was 80% female with an average age of 39.4 years. Psychotherapy resulted in lower mean depression scores compared with no treatment (ES = 0.93). No differences were found by sex. Gloaguen et al (36) conducted a meta-analysis of 48 RCTs to evaluate the effectiveness of CT for treatment of major depression or dysthymic disorder. They found that CT was more effective than antidepressants, and there were no differences by sex.

Three articles examined the effectiveness and safety of SSRIs and SNRIs in women, and all were post-hoc analyses of pooled data (Table 3). Two studies examined the effect of SSRIs on level of depression (38,39). Khan et al (39) found rates of response (65% vs 40%, *P* < .001) and remission (45% vs 14%, *P* < .001) greater for women taking SSRIs than placebo. Entsuah et al (38) found higher rates of depression absence (31% vs 20%) and remission (34% vs 24%) among women taking SSRIs compared with those taking placebo (*P* < .05). Among participants aged 40 years or younger, differences in rates of remission and absence of depression between those taking SSRIs and those taking placebo did not reach statistical significance (38). Three studies examined the effects of different SNRIs on depression among women (38-40) and found greater response and remission rates among women taking SNRIs compared with those taking placebo (38-40).

## Discussion

Our systematic review of the literature identified several gaps in knowledge of screening, detection, and treatment of depression among nonpregnant women of reproductive age. Current USPSTF screening guidelines do not recommend universal screening for depression among adults unless staff supports are in place to diagnose, treat, and follow up patients (17). It is unclear what percentage of clinical practices serving nonpregnant women of reproductive age fit these criteria. The percentage of women who are screened and the percentage of providers who screen nonpregnant women for depression are largely unknown. The limited data available suggest low screening rates, although prevalence of depression is high. Several validated screening tools exist and perform equally well in primary care settings. However, we found no studies examining the performance of the screening tools specifically among nonpregnant women of reproductive age.

Additionally, no studies were found that examined the effect of depression screening on diagnosis, treatment, and outcomes specifically among nonpregnant women of reproductive age. Studies suggest that screening in high-risk populations may be effective in clinician recognition of depression and patient receipt of treatment (29). Women of reproductive age may be considered high risk, especially low-income women attending public family planning and OB/GYN clinics, where, in 3 studies from different parts of the United States, 19% to 48% of women screened positive for moderate to severe or clinically relevant depression (41-43). Engaging low-income women in treatment can improve depressive symptoms; however, it takes considerable time and resources, and engagement is difficult to achieve, even when treatment is free and child care and transportation are provided (44).

Various treatment options exist for depressed women, including exercise, psychotherapy, and pharmacotherapy. In England, exercise is a first-line therapy (45), but not in the United States. All treatments reviewed performed better than placebo; however, treatment response rates were low. Further research is needed to identify whether combining treatments can further improve effectiveness.

In summary, although studies have documented high rates of depression among women of reproductive age and significant detrimental health effects for them and their families, how to engage women in effective treatment and how to create systems of affordable and acceptable care remain future challenges. Recent changes in health insurance through national health care reform may provide insurance coverage for more people with mental illnesses. Monitoring access and use among women of reproductive age will help evaluate whether this policy improves mental health in the United States.

## Figures and Tables

**Table 1:** Research Question, Inclusion Criteria, and Population Included,^a^ Systematic Review of Articles on Depression Screening and Treatment Among Nonpregnant Women of Reproductive Age in the United States, 1990-2010

**Inclusion Criteria**	**Population Included**	Studies Meeting Inclusion Criteria (Type)
**1. What are the current national clinical practice recommendations and guidelines for depression screening?^b^**		
Meets criteria for inclusion in the National Guideline Clearinghouse (http://www.guideline.gov/about/inclusion-criteria.aspx) Additional requirements included: Was developed by a national organization. Provides detailed information about the methods used to search, collect, and select the evidence (either in the original guideline, or in an associated evidence review). Provides details about the methods used to assess the quality of the literature (eg, either rating or grading the literature that the recommendations are based on, providing evidence tables and detailed discussion of the underlying evidence). Makes clear recommendations. Includes the target population. Specific reference to the target population is not necessary to meet this inclusion criterion. For example, guidelines for the general adult populations would be included as they are assumed to be relevant to the target population (women of reproductive age who are not pregnant).	Adult men and women	5 (guidelines)
**2. What are the prevalence and predictors of screening?**		
Describes a study that screened patients for depression or a survey of clinicians' reports or medical record review of screening for depression.	Adult women and women seen by obstetrician/gynecologists	4 (individual studies)
**3. How well do screening tools detect depression?^b^**		
Describes a validated screening tool for depression in adult primary care populations.	Adult men and women	1 (meta-analysis)
**4. Does screening lead to diagnosis, treatment, and improved outcomes?^b^**		
Presents data related to the rates of depression diagnosis and treatment among patients screened for depression compared with patients not screened.	Adult men and women	3 (2 meta-analyses, 1 systematic review)
**5. What are the most effective treatment methods?**		
Examines the effect of exercise, psychotherapy, SSRI, or SNRI^c^ on depression.	Adult men and women from the general population with results presented separately for women, or gender evaluated as an effect modifier	8 (5 meta-analyses, 3 post-hoc analyses of pooled data)

Abbreviations: SSRI, selective serotonin reuptake inhibitor; SNRI, selective norepinephrine reuptake inhibitor.

a All studies and reports were full-length articles published in the English language, between 1990 and 2010.

b Given the lack of studies reporting data separately for women, articles for the general US population were included.

c Articles on SSRIs and SNRIs were limited to those published between 2000 and 2010.

**Table 2:** National Guidelines and Recommended Screening Tools for Depression Screening in Adults, 2010

**Organization, Year**	**Recommendation**	**Screening Tool**
US Preventive Services Task Force, 2009 (17)	Screen adults for depression when staff-assisted depression care supports are in place to ensure accurate diagnosis, effective treatment, and follow-up. Do not routinely screen adults when staff-assisted care supports are not in place. There may be considerations to support screening in an individual patient.	Several screening tools exist. Insufficient evidence to support 1 method over another.
American Academy of Family Physicians, 2010 (19)	Screen adults for depression only when staff-assisted depression care supports are in place to ensure accurate diagnosis, effective treatment, and follow-up. Recommends against routinely screening adults for depression when staff-assisted depression care supports are not place. There may be considerations that support screening for depression in an individual patient.	Clinicians may choose the method that best fits their personal preference, the patient population served, and the practice setting.
US Department of Veterans Affairs, 2009 (18)	Annual screening of adult patients seen in primary care.	Screen using a standardized tool such as the PHQ-2. Use PHQ-9 as an aid for diagnosis, measurement of symptom severity, and to assess treatment response.
American College of Obstetricians and Gynecologists, 2007 (16)	Does not advocate for or against routine screening. Screening can consist of a written questionnaire, or clinician may ask if other symptoms are present. Clinicians should provide follow-up care for women identified with depression or refer elsewhere for care. Referral is recommended for women with depression with suicide risk or psychotic symptoms, those with bipolar disorder, depressed adolescents, patients who fail to respond to treatment, substance abusers, or if clinician is not comfortable treating patient.	Suggests PHQ-2 as an example of 1 of many valid screening tools.
American Academy of Pediatrics, 2010 (20)	Recommends screening mothers for depression at the 1-, 2-, 4-, and 6-month well-child visits and beyond the postpartum period. Based on the severity of the depression score, pediatricians should provide reassurance, supportive strategies, and referral for specific interventions.	Edinburgh Postnatal Depression Scale for postpartum depression and PHQ-2 to assess depression outside of the postpartum period.

Abbreviations: PHQ-2, Patient Health Questionnaire, 2-question version; PHQ-9, Patient Health Questionnaire, 9-question version.

**Table 3:** Meta-analyses and Post-hoc Analyses of Pooled Data Evaluating Effectiveness of Treatments for Depression Among Women of Reproductive Age

**Treatment**	**Description**
**Exercise**	
Authors, year	North et al, 1990 (35)
Type of analysis	Meta-analysis
Studies included: design(s); total number/number women only; years studies published	RCT, non-RCT comparative, 1 group pre-post, matching groups, convenience sample groups, and pre-test/post-test studies with any measure of depression as dependent variable; 80/16; English language; published or scheduled for publication on or before June 1, 1989.
Population of interest and exclusion criteria	Studies of any reported depression including mood disorders, psychogeneous or endogenous types of depression, primary or secondary
Outcome, intervention, comparison group, and covariates	Outcome: Level of depression; intervention: aerobic and resistance exercise; comparison groups: no treatment, wait-list, psychotherapy, enjoyable activity, relaxation, less exercise, anaerobic exercise, exercise and psychotherapy; covariates: source of subjects, group assignment, degree of internal validity, initial level of depression, age, sex, exercise duration per episode, type, frequency and intensity, additional therapy, health status.
Results	The overall ES^a^ of exercise on depression was −0.53 ± 0.85.Significant main effects were found for source of subjects (medical/psychological patients > students/citizens); source of study (published > unpublished); purpose of exercise (medical rehabilitation > general health or psychological rehabilitation); health status (sicker > healthier); type of exercise (weight training > aerobic); duration (longer > shorter). No significant moderating effects found for group assignment, internal validity, sex, age, or depression diagnosis.
Limitations	Included non-RCT studies and studies with depression as secondary diagnosis to another mental health condition.
Authors, year	Craft and Landers, 1998 (33)
Type of analysis	Meta-analysis
Studies included: design(s); total number/number women only; years studies published	RCT, non-RCT comparative and pretest/posttest studies with measure of depression as dependent variable; 37/4; published or scheduled for publication on or before November 1996.
Population of interest and exclusion criteria	2,158 people with depression as primary diagnosis or secondary diagnosis to another mental health condition; excludes studies on depression as a result of physical health problem.
Outcome, intervention, comparison group, and covariates	Outcome: level of depression; intervention: aerobic and resistance exercise; comparison groups: wait list, group or individual therapy, behavioral interventions; covariates: initial level of depression; age; sex; exercise duration per episode, type, frequency, and intensity; additional therapy.
Results	Compared with wait-list controls, people who exercised were ES 0.77 (95% CI, −1.08 to −0.47) less depressed than individuals on a wait list. Exercise was as beneficial as group/individual therapy and behavioral interventions.Significant main effects were found for initial level of depression (moderate to severe group > mild to moderate group); source of study (published > unpublished); primary versus secondary depression; no significant moderating effects were found for sex, age, or exercise type, duration, frequency, or intensity on the relationship between exercise and depression. Individuals who exercised 9–12 weeks were less depressed than those who exercised ≤8 weeks.
Limitations	Included non-RCT studies; included depression as secondary diagnosis to another mental health condition; limited generalizability because of exclusion criteria; measures of level of depression not stated.
Authors, year	Rethorst et al, 2009 (34)
Type of analysis	Meta-analysis
Studies included design(s); total number/number women only; years studies published	RCTs only; 58/7; 1981-2005.
Population of interest and exclusion criteria	2,982 people with depression (574 clinical, 2,408 nonclinical); depression associated with physical or psychological illness excluded
Outcome, intervention, comparison group and covariates	Outcome: level of depression; intervention: moderate to vigorous aerobic or resistance exercise; comparison group: no treatment or wait list, secondary comparison groups: psychotherapy and antidepressant medication; covariates: depression type, intervention duration, exercise type, frequency, bout duration, sex, methodological characteristics, treatment adherence, dose response.
Results	Overall ES of −0.80 indicates participants in the exercise treatment had significantly lower depression scores than controls. Exercise was more effective among clinically depressed participants (ES = −1.03) than nonclinical samples (ES = −0.59). Aerobic and resistance exercises were equally effective. No significant differences in the effect of exercise compared with psychotherapy or antidepressant medication. No significant moderating effects found for sex on the relationship between exercise and depression.
Limitations	Limited generalizability because of exclusion criteria.
**Cognitive therapy**	
Authors, year	Gloaguen et al, 1998 (36)
Type of analysis	Meta-analysis
Studies included: design(s); total number/number women only; years studies published	RCTs only; 48/4; Published studies and those presented at international congresses, 1977–1996
Population of interest and exclusion criteria	2,765 people with major depression or mild/moderate dysthymia; excluded psychotic and bipolar disorder
Outcome, intervention, comparison group and covariates	Outcome: level of depression assessed by BDI; intervention: cognitive therapy; Comparison group: waiting list or placebo, antidepressant medication, behavioral therapy, other psychotherapeutic treatment (psychodynamic therapies, interpersonal therapies, nondirective, supportive, relaxation and alternative bibliotherapy); covariates: BDI scores, sex, age.
Results	Cognitive therapy was significantly better than waiting-list or placebo (*P* <.001), antidepressants (P < .001), and a group of miscellaneous therapies (*P* <.001). No covariates modified effect size in multivariable analysis.
Limitations	Limited to published articles and studies presented at international congresses. Limited generalizability because of exclusion criteria.
**Psychotherapy**	
Authors, year	Robinson et al, 1990 (37)
Type of analysis	Meta-analysis
Studies included design(s); total number/number women only; years studies published	Studies comparing treatment to no treatment or different types of therapy; excluded case histories and pre-post designs and treatments without a prominent verbal component, and marriage/family therapy; 58/unknown; Mean percentage of female clients per study was 80%; range: 50%–100%; psychological abstracts 1976-1986 and relevant journals 1985-1986.
Population of interest and exclusion criteria	People with depression either meeting formal diagnostic criteria or screening positive; excluded subjects described in more general terms or by other specific diagnoses; studies examining inpatients or children and adolescents also excluded; mean age: 39 years, range: 19-71
Outcome, intervention, comparison group, and covariates	Outcome: depression symptoms; assessed by multiple different validated screeners for depression, general mental health and functioning; intervention: 1 of 4 types of therapies: 1) cognitive, 2) behavioral, 3) cognitive-behavioral, 4) general verbal, 7 mean weeks of treatment and 8.7 mean number of sessions; comparison: no treatment (n = 46 studies), wait list (n = 29 studies), placebo (n = 9 studies); covariates: sex and age, weeks of treatment, number of sessions.
Results	Psychotherapy was more effective than no treatment (ES = 0.73 at posttreatment and 0.68 at follow-up, average 13 weeks after treatment) and wait list (ES = 0.84) (*P* < .05 for all). In the 29 studies using outcome measures specific to depression, psychotherapy was more effective than wait list (ES = 0.93, *P* < .05). No differences in effect sizes found by sex, age, weeks of treatment, or number of sessions.
Limitations	Included non-RCTs; limited to published articles and abstracts presented at conferences.
**SSRIs/SNRIs**	
Authors, year	Entsuah et al, 2001 (38)
Type of analysis	Post-hoc analysis of pooled data
Studies included design(s); total number/number women only; years studies published	Included placebo controlled double-blind, active-controlled phase 2,3, or 5 trials; 8/0; 62%-65% of participants female, depending on intervention type; published or reported 1992 to 1998.
Population of interest and exclusion criteria	2,045 people meeting DSM-III or IV criteria for major depressive disorder and ≥20 on HAM-D-21 or 25 on Montgomery Asberg Depression Rating Scale; age range 18-83 years; inpatients (n = 67) and outpatients (n = 1,977); excluded pregnant, lactating; significant history of cardiovascular, renal, hepatic, or seizure disorders; abnormal physical examination or electrocardiogram; history of alcohol or drug abuse; use of investigational or antipsychotic drugs in last 30 days, monoamine oxidase inhibitors within 14 days, or antidepressants anxiolytics or sedative-hypnotic drugs within 7 days.
Outcome, intervention, comparison group and covariates	Outcome: depression absence, response or remission to treatment, measured by score of 0 on depressed mood item of HAM-D-21; score ≤7 on HAM-D-17; and ≥50% decrease in score on HAM-D-21, respectively; intervention: venlaflaxine (n = 865); SSRI (n = 757); 6-12 weeks; comparison group: placebo (n = 450); covariates: age, sex
Results	For both men and women, both venlaflaxine and SSRIs were significantly more effective in depression absence, response, and remission than placebo. At 8 weeks, rates of remission among women treated with venlaflaxine were higher (45%) than those among people receiving SSRIs (34%) or placebo (24%), *P* < .001. Rates of response with venlaflaxine (65%) were higher than placebo (43%), *P* < .001. Rates of absence of depressed mood among women receiving venlaflaxine (37%) or SSRI (31%) were higher than placebo (20%), *P* < .003. Among participants ≤40 years (men and women combined), rates of remission (*P* < .001) and absence of depression (*P* < .001) were higher with venlafaxine than placebo. Differences between SSRIs and placebo were not statistically significant among participants ≤40 years. No effect of age or sex on effectiveness in depression absence, remission, or response to venlaflaxine or SSRIs.
Limitations	Limited generalizability; disproportionately large number of patients treated with fluoxetine in SSRI group; no studies examined setraline or citalopram; no information on menopausal status.
Authors, year	Khan et al, 2006 (39)
Type of analysis	Post-hoc analysis of pooled data.
Studies included design(s); total number/number women only; years studies published	Double-blind RCTs, phase 2, 3, and 4; 15/0; conducted 1996–2003.
Population of interest and exclusion criteria	323 people with depression; 177 (55%) women with depression; n = 80 on placebo; n = 71 on SSRI; n = 26 on SNRI; excluded people with severe illness, suicidal patients, and patients with concomitant disorders.
Outcome, intervention, comparison group and covariates	Outcome: level of depression assessed by response and remission rates from HAM-D-17 scores; intervention: SSRI (fluoxetine, paraxetine CR, sertaline, citalopram, excitalopram), SNRI (venlaflaxine ER); comparison group: placebo; covariates: baseline depression score and sex.
Results	Response rates greater for women taking SSRIs (64.8%) and SNRIs (69.2%) than placebo (40%) (*P* < .001). Remission rates higher in women taking SSRIs (45.1%) and SNRIs (46.2%), than placebo (13.8%) (*P* < .001). Women taking SSRIs had ES 0.82, and women taking SNRI had ES 0.76. Effect of SSRIs greater in women than men (*P* = .001). No difference between women and men taking SNRI.
Limitations	Duration of medication use not stated; limited generalizability because of exclusion criteria.
Authors, year	Kornstein, et al, 2006 (40)
Type of analysis	Post-hoc analysis of pooled data.
Studies included design(s); total number/number women only; years studies published	Randomized, multicenter, double-blind, placebo-controlled trials; 7/0; 2002-2005.
Population of interest and exclusion criteria	N = 1622 people with major depressive disorder defined as HAM-D-17 score >15; 1,062 women n = 578 duloxetine; n = 484 placebo; mean age 41 years; excluded people with current and primary Axis I disorder other than depression, an Axis II disorder, lack of response to ≥2 courses of antidepressant therapy, serious medical illness, risk of suicide, history of substance abuse in last year, positive drug screen.
Outcome, intervention, comparison group and covariates	Outcome: level of depression; intervention: duloxetine (40, 60, 80, and 120 mg/day); comparison group: placebo; covariates: sex, study design characteristics, methodological characteristics, study duration (7-9 weeks).
Results	For women, duloxetine was more effective than placebo in HAM-D-17 (ES = 0.22, P <.001), CGIS (ES = 0.19, P <.001), and PGI-I (ES = 0.30, P <.001) measures. Significantly greater improvement in VAS pain scores in duloxetine-treated compared with placebo-treated women.
Limitations	Duration of treatment 7-9 weeks; menopausal status unknown; generalizability limited because of exclusion criteria.

Abbreviations: RCT, randomized controlled trial; ES, effect size; CI, confidence interval, BDI, Beck Depression Inventory; NR, not reported; SSRI, selective serotonin reuptake inhibitor; SNRI, selective norepinephrine reuptake inhibitor; HAM-D, Hamilton Rating Scale for Depression; CGIS, Clinical Global Impressions-Severity; PGI-I, Patient Global Impression of Improvement scale; VAS, visual analogue scale.

a ES = standardized mean difference between intervention and control groups.

## Appendix. Free-Text Search Terms Related to Screening and Treatment of Depression, Population-Based Surveys, and Study Types Used in Electronic Searches

**Table:**

**MeSH**	**Free-Text**
**Disease-specific-related terms**	
Depression Depressive disorder	Side effects Adverse events Adverse effects
**Screening and treatment-related terms**	
Mass screening Psychotherapy Exercise Exercise therapy Physical fitness Utilization Computers Relaxation Music Bibliotherapy Pharmacotherapy	Screening Screening trends Diagnosis Exercise Prevention Physical activity ComputerInternet Positive psychology Self-helpBehavioral activation Light therapy Treatmen tDisease management Disease prevention Selective serotonin reuptake inhibitors Selective norepinephrine reuptake inhibitors
**Other**	
Cross-sectional survey Health surveys Review Meta-analysis Guideline Randomized controlled trial	NHANES NHIS BRFSS MEPS NAMCS Population survey Systematic review Meta-analysis Clinical practice guideline Evidence-based guidelines Standards

Abbreviations: NHANES, National Health and Nutrition Examination Survey; NHIS, National Health Interview Survey; BRFSS, Behavioral Risk Factor Surveillance System; MEPS, Medical Expenditure Panel survey; NAMCS, National Ambulatory Medical Care Survey.
